# The Mindset of Intelligence Is Not a Contributor of Placebo Effects in Working Memory Training

**DOI:** 10.3389/fpsyg.2021.712309

**Published:** 2021-11-04

**Authors:** Peibing Liu, Xin Zhang, Renlai Zhou

**Affiliations:** ^1^Department of Psychology, Nanjing University, Nanjing, China; ^2^Smart Home Solution BU of Innovation Business Group, TCL Industries Holdings Co., Ltd., Huizhou, China

**Keywords:** working memory training, fluid intelligence, placebo effects, mindset of intelligence, transfer effect

## Abstract

Whether working memory training is effective in enhancing fluid intelligence remains in dispute. Several researchers, who doubt the training benefits, consider that placebo effects may be the reason for positive training gains. One of the vital variables that may induce the placebo effect is the mindset of intelligence. In this article, we provide a test of whether the mindset of intelligence leads to placebo effects in working memory training. Participants were overtly recruited and allocated to the growth mindset group or the fixed mindset group by Theories of Intelligence Scale scores. A single, 1 h session working memory training is the cue to introduce the placebo effects. During pre/post-testing, all participants completed tasks measuring working memory capacity (near transfer) and fluid intelligence (far transfer). Our findings show no significant difference between the two groups in both tasks. Therefore, these results suggest that the placebo effect does not exist in this study, which means individuals' mindset of intelligence may not be a contributor to the placebo effect in 1 h working memory training. This research will further help to clarify the mechanism of the placebo effect in working memory training.

## Introduction

Working memory is a cognitive system that plays a crucial role in keeping things in mind while performing complex attentional-cognitive control activities such as goal-directed behavior, reasoning, decision-making, comprehension, and learning (Kane and Engle, 2002; Holmes et al., 2009; Baddeley, 2010; Shahar et al., 2018). From this perspective, working memory training is assumed to improve not only working memory capacity but also a battery of related abilities. Several studies have verified the assumption that the training can enhance attention (Chein and Morrison, 2010; Kundu et al., 2013), decrease attention deficit hyperactivity disorder (ADHD)-related symptoms (Klingberg et al., 2002, 2005), and strengthen reading or language comprehension (Carretti et al., 2013, 2014; Artuso et al., 2019). In 2008, a study found a promising result, short-term working memory training can improve the fluid intelligence of healthy adults, to support that fluid intelligence is trainable (Jaeggi et al., 2008; Sternberg, 2008). Building on this initial research, more studies on working memory training on fluid intelligence have accumulated (Jaeggi et al., 2011, 2014; Hardy et al., 2015). Fluid intelligence refers to the ability to solve novel, abstract problems through insight into complex relationships without relying on previous knowledge experience (Cattell, 1963). Fluid intelligence is not only the basis for other cognitive abilities but also plays a key role in how we solve problems in daily work and life and how we adapt to new situations (Sternberg and Gastel, 1989). Although many studies have reported that the near-transfer effect (i.e., increased working memory ability) is statistically significant and lasts for several months, some controversial results remain on the far-transfer effect (i.e., improvement in other abilities), especially on the transfer to fluid intelligence (Colom et al., 2010, 2013; Owen et al., 2010; Chooi and Thompson, 2012; Bastian and Oberauer, 2013; Redick et al., 2013; Sprenger et al., 2013; Thompson et al., 2013; Bastian and Eschen, 2016; Lawlor-Savage and Goghari, 2016; Clark et al., 2017). Whether working memory training can increase fluid intelligence still lacks consistent evidence.

Considering the argument, the role of placebo effects in early positive findings, expectations may lead to post-training fluid intelligence gains, is of a central concern (Shipstead et al., 2012; Slagter, 2012; Melby-Lervåg and Hulme, 2013; Redick et al., 2013). Placebo effects are psychophysiological changes caused by the symbolic meaning of treatment rather than specific pharmacological or physiological properties (Brody, 1980; Stewart-Williams and Podd, 2004). It is well-known that in drug trials, the control group takes a placebo pill (which looks the same as the experimental group) to promise both to have the same anticipation for the pills. In medical practice, one reason for the improvement experienced by a patient after treatment is the confidence of the patient in the healer or the drug is so strong that the psychological effect of the relief alleviates their condition (Zhang et al., 2011). The improvement is not caused by the treatment itself. It is the belief and expectation (the treatment will work) that lead to the placebo effect. Psychological intervention, including working memory training, should also pay attention to the difference in expectations between groups (Boot et al., 2013). Foroughi et al. (2016) published an infusive report confirming the placebo effects in 1 h working memory training. In this research, the placebo group was recruited with a flyer that overtly advertised the cognitive enhancement effect of working memory training; the control group was recruited covertly with a visually similar flyer. During the 1 h training session, the results showed that fluid intelligence was significantly improved in the placebo group rather than the control group. These results suggest that the observed effects are due to overt recruitment (reveal the objective to induce expectancies), which challenges the true efficacy of working memory training.

It is worth noting that, in the study of Foroughi et al. (2016), the average Theories of Intelligence Scale (TIS) scores of the placebo group were significantly higher than the control group. The TIS measures the mindset of intelligence, which reflects beliefs regarding the malleability of intelligence. Students can hold different “theories” about their intelligence (Dweck, 2000). Some students who have a fixed mindset believe that their intelligence is unchangeable (an entity theory). Others who have a growth mindset believe that their intelligence can be developed through effort and persistence (an incremental theory). Individuals who have high TIS scores may consider that their intelligence can be improved. However, Foroughi et al. only attributed positive post-test outcomes to the recruitment methods. The role of the mindset of intelligence in placebo effects is ambiguous.

According to the mindset theory (Dweck, 2000), students with a growth mindset have many benefits, including adherence to learning goals rather than performance goals (Richard and Pals, 2010), positive effort belief (Tempelaar et al., 2015), and holding effort and positive strategies (Ommundsen et al., 2005). These also lead to better academic achievement (Claro et al., 2016). However, this theory has been discredited and inconsistent empirical findings were found (Dommett et al., 2013). A recent meta-analysis (Sisk et al., 2018) found that the relationship between mindset interventions and academic achievement was non-significant, which did not support Dweck's claim. This also suggests that more related research is needed. Therefore, our study (the mindset of intelligence is a critical variable) can also be seen as an answer to this need.

Several studies emphasize the influence of individual differences on training results (Jaeggi et al., 2014; Guye et al., 2017), among which mindset of intelligence is an essential factor. Individuals who think their intelligence is malleable show a greater transfer effect in training than those who think intelligence can hardly be changed through effort (Jaeggi et al., 2014). Clinical empirical studies have supported that mindset is one of the factors inducing the placebo effect. Mindset can lead to the attention and motivation of patients and affect the subjective and objective measurements of health and well-being (Crum and Zuckerman, 2017). Moreover, according to the study on stress, diet, and exercise, the mindset was related to mental and physical well-being, including blood pressure, weight loss, and cortical and hormonal responses (Crum and Langer, 2007; Crum et al., 2013; Crum and Zuckerman, 2017). Before being informed of the disease and treatment information, patients already have a certain mindset, which can interpret the information reception, affect subsequent expectancies, and induce the placebo effect (Zion and Crum, 2018). Similarly, the mindset of intelligence could be a contributor to placebo effects in working memory training. That is, the variable, mindset of intelligence, may have confounded the results reported in the Foroughi et al.'s (2016) study. Therefore, empirical research is needed to examine whether overt recruitment or the interaction between overt recruitment and mindset of intelligence contributes to the placebo effects in working memory training.

To answer this question, we adopted the same recruitment paradigm, procedure, and sample size as in the study by Foroughi et al. (2016). Additionally, we ensured no difference in TIS scores between the placebo group and the control group (Zhang et al., 2019). However, we found no improvement of fluid intelligence in either group, which failed to replicate the findings of the Foroughi et al.'s (2016) study. These results ruled out the separate role of overt recruitment in positive post-test outcomes, leading us to wonder whether the mindset of intelligence is the cause of the placebo effect. This study aims to illustrate these questions.

For this aim, in this study, we recruited both groups overtly by advertising fluid intelligence improvement. Importantly, before the formal experiments, participants filled in the TIS and were divided into the growth mindset group (higher TIS scores) and the fixed mindset group (lower TIS scores). Therefore, if the growth group rather than the fixed group has task performance enhancement (placebo effect), it would support that mindset of intelligence is the contributor to task performance. Three different versions of adaptive working memory training tasks were used in the 1 h cognitive training session. In addition to the fluid intelligence (far-transfer effect), we also used a 2-back task to measure the near-transfer effect.

## Materials and Methods

### Participants

**Figure 1:**
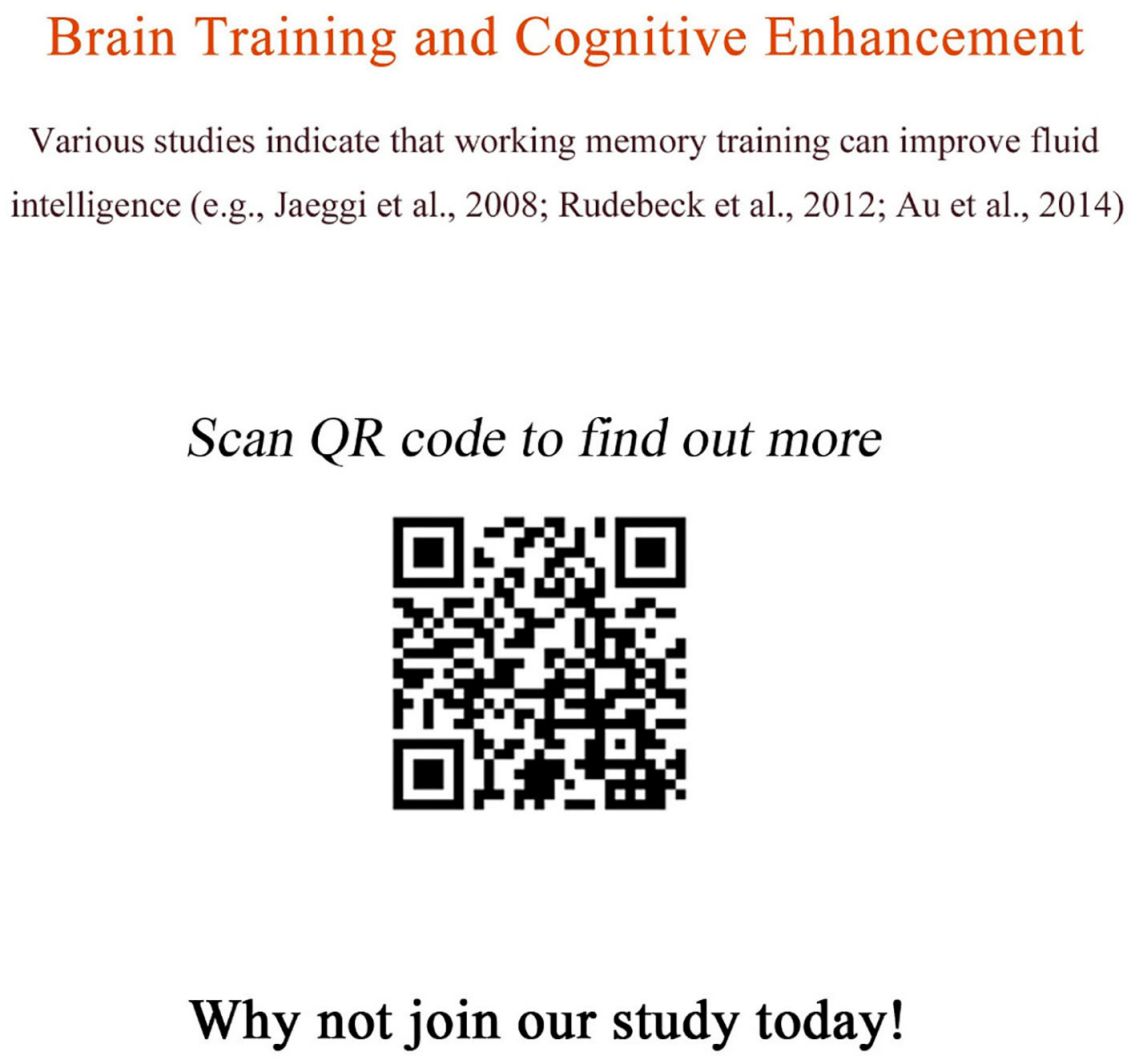
Poster (the original poster was in Chinese).

Participants were recruited at Nanjing University *via* an overt advertisement poster stating that “Various studies indicate that working memory training can improve fluid intelligence” (Figure 1). Participants are all adults aged between 18 and 25 years, right-handed, in good health, and not taking any drugs. All participants provided informed, written consent before the formal experiment.

We recruited a total of 50 participants and divided them into two groups of 25 persons, the same size as Foroughi et al.'s (2016) study, based on their TIS scores. The specific recruitment process is as follows: we first show our recruitment posters. If students are interested in our experiment, they can scan the QR code on the posters to fill in a questionnaire, which is the TIS. After the statistical analysis on the scale scores of participants, we will invite participants with scores >35 and <20 to participate in our formal experiments. The TIS scores in the growth mindset group are >35 (M = 36.6, SD = 2.26); in the fixed mindset group, the TIS scores are <20 (M = 16.92, SD = 2.93). Table 1 provides detailed information on participants. The two groups did not demonstrate any significant difference in gender ratio [χ^2^ (1, *N* = 50) = 0.104, *P* = 0.747, Cramer's *V* = 0.046] or in average age [*t*_(48)_ = 1.393, *P* = 0.170, Cohen's *d* = 0.39, 95% CI = −0.337, 1.857].

**Table 1:** Characteristics of participants.

	**Growth mindset group (*N* = 25)**	**Fixed mindset group (*N* = 25)**	**Group differences (*p*-value)**
Gender (male: female)	7:18	6:19	0.747
Age (years; M ± SD)	20.76 ± 2.17	20.00 ± 1.66	0.170
TIS scores	36.6 ± 2.26	16.92 ± 2.93	<0.001

### Procedure Overview

**Figure 2:**
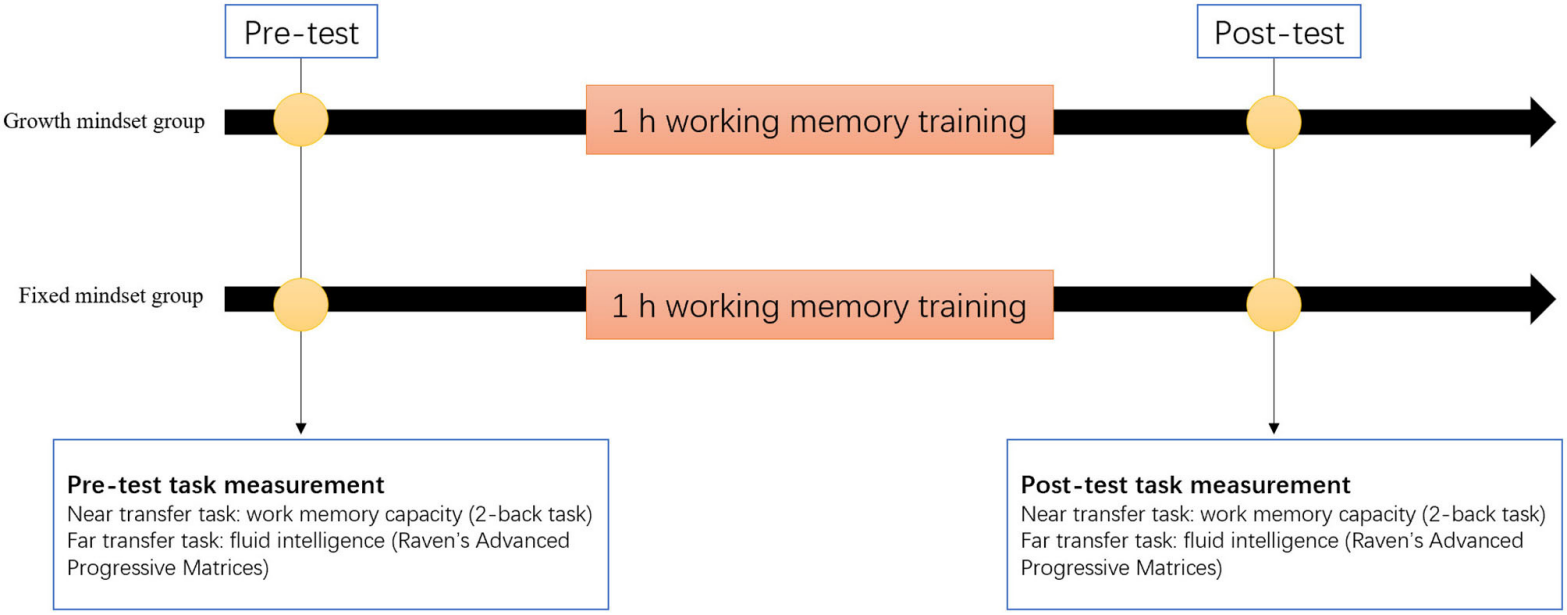
Procedure for the training study.

Figure 2 describes the procedure for the study. The experiment was divided into three parts: pretest, working memory training, and post-test. All participants attended the pretest and post-test tasks (2-back task measuring near-transfer effect; Raven's Advanced Progressive Matrices (RAPM) measuring far-transfer effect). A single, 1 h session working memory training was conducted between the pretest and post-test. Similar to Foroughi et al. (2016), the reason for choosing an hour as training duration is that training time should be adequate (roughly 20 sessions, each lasting 30–60 min) to make cognitive training effective (Shipstead et al., 2012), so the positive outcomes from 1 h training must be due to placebo effects. At the end of the experiment, all participants were compensated with money.

### Training Tasks

**Figure 3:**
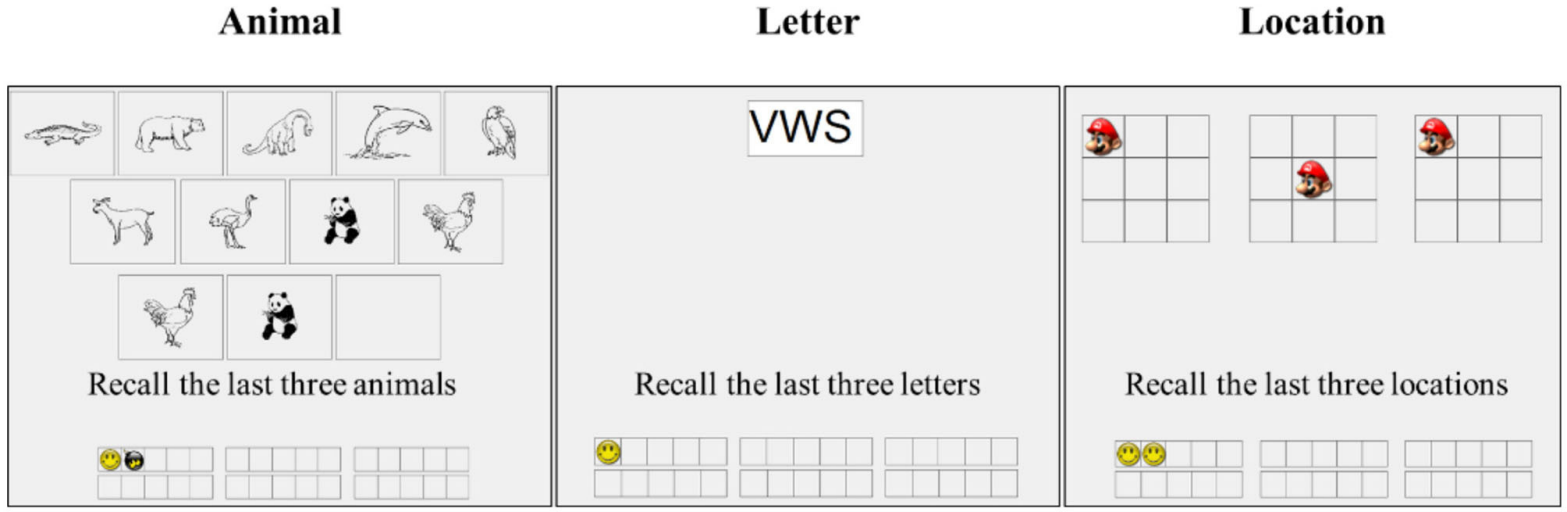
Demonstration of three training tasks.

After the pretest, participants completed a computerized working memory training. We used three different versions of adaptive working memory training tasks, including three kinds of memory materials: animals, letters, and positions (Figure 3). Based on the classical running memory span task, many studies have previously adopted the training tasks in cognitive training research (Zhao et al., 2011; Wang et al., 2014; Chen et al., 2018). We will take the animal training task as an example to explain the operation of training tasks in detail. In the animal training task, animals are different and presented in the center of the screen in sequence. In each trial, the number of animals varied randomly from 5 to 7, 9, and 11, and participants were asked to remember the last three animals presented in this trial. It is worth noting that participants could not predict the number of animals that would appear in each trial, they were not told, so they had to update the memory items constantly. It would train the working memory updating abilities. The other two training tasks are similar to the animal training task: participants must report the last three letters of the alphabet training task and the last three animation positions in the position training task. Each training task consists of 30 trials, which are divided into six blocks with five trials each. At the beginning of the training, the duration of each stimulus was 1,750 ms. If participants correctly reported three or more trials in this block, the duration would be decreased by 100 ms in the next block. In this study, participants completed each training task two times in a randomized order, which lasted approximately an hour.

### Transfer Measurements

#### Working Memory Capacity (Near Transfer)

We used a computerized 2-back task to assess the near-transfer effect in working memory training. In this task, participants were asked to press key “F” if the currently presented item was the same as the item presented two steps earlier; press “J” if not. The matching and mismatching stimuli were both presented 50% in this task. A “+” was always presented in the center of the screen, and a series of numbers, ranging from 0 to 9, will appear at the top, bottom, left, and right of the “+.” Participants are required to ignore the verbal information and judge whether the digital space position of the current number matches the target stimulus. Numbers and their spatial positions are both random. Each trial consisted of a fixation (200 ms), a blank screen interval (1,300 ms), target (200 s), and reaction time (until response or until 2,500 ms). Reaction time and accuracy were included in the analysis.

#### Fluid Intelligence (Far Transfer)

One of the far-transfer effects that researchers are most concerned with is fluid intelligence. In this study, we utilized Raven's Advanced Progressive Matrices (RAPM), commonly used in adult intelligence research, to assess fluid intelligence change (Raven et al., 1998). Referring to Jaeggi et al. (2008), we used parallel forms for the pretest and post-test by dividing the RAPM test into even and odd items.

## Results

All analyses were conducted using mixed-effects linear regression with restricted maximum likelihood.

### Training Effects

**Figure 4:**
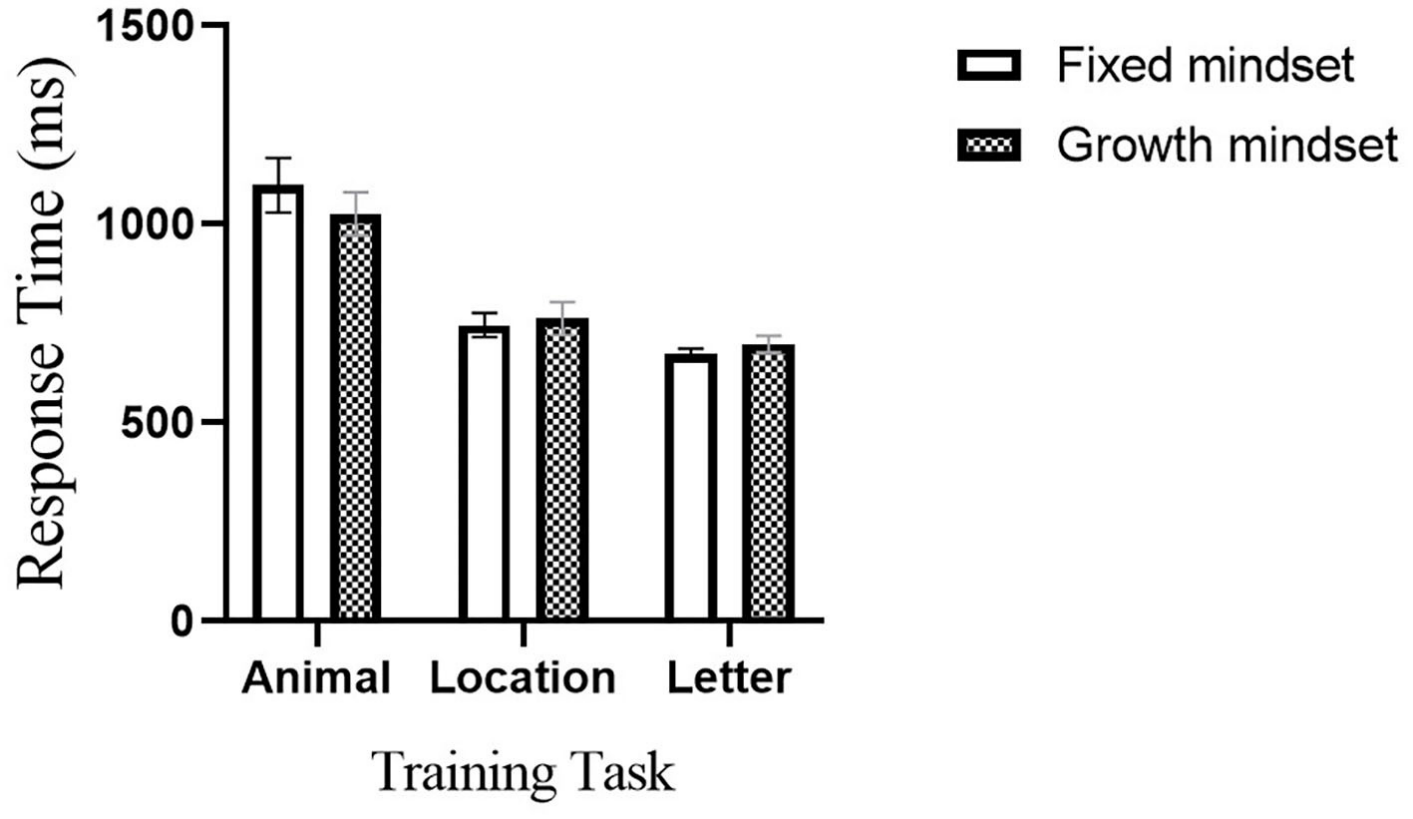
The difference between the fixed and growth groups in working memory training tasks (animal, location, and letter).

We compared whether there were differences in training tasks between the two groups (Figure 4). The performance of participants in training tasks can be measured by the task difficulty level they eventually reach. The time interval between adjacent stimuli presents the difficulty of the training task. The shorter the time interval between stimuli, the more difficult the task is. The baseline interval was 2,250 ms. The maximum level of difficulty that participants attained did not differ between the two groups: *B* = 72, SE = 87.64, *t*_(48)_ = 0.822, *P* = 0.416, *b* = 0.24 for animal task; *B* = −16, SE = 51.90, *t*_(48)_ = −0.308, *P* = 0.759, *b* = 0.09 for location task; and *B* = −24, SE = 24.77, *t*_(48)_ = −0.969, *P* = 0.338, *b* = 0.30 for letter task, respectively.

### Transfer Effects

#### Two-Back Task (Near Transfer)

**Figure 5:**
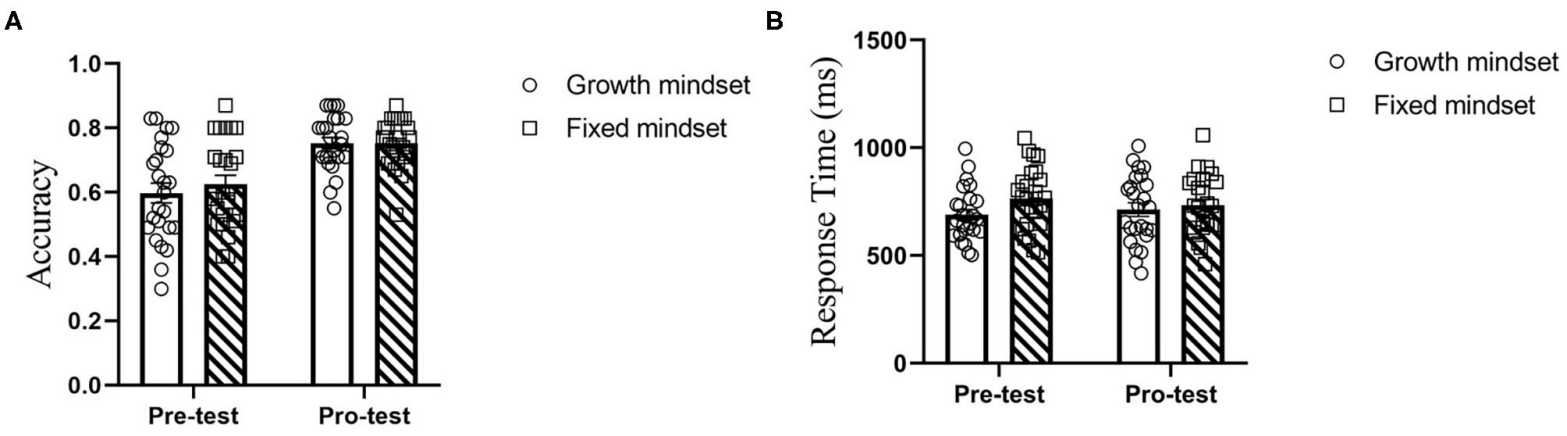
The difference between the growth group and the fixed group in the 2-back task [accuracy **(A)** and response time **(B)**].

We calculated the accuracy and reaction time difference between the two groups in 2-back task from pretest to post-test. At the pretest, the two groups did not significantly differ in accuracy [*B* = −0.27, SE = 0.41, *t*_(48)_ = −0.670, *P* = 0.506, *b* = 0.19] or reaction times [*B* = −75.07, SE = 38.05, *t*_(48)_ = −1.973, *P* = 0.054, *b* = 0.58]. After the training, there was still no significant difference in accuracy [*B* = 0.0004, SE = 0.02, *t*_(48)_ = 0.018, *P* = 0.986, *b* = 0.005] or reaction times [*B* = −19.72, SE = 42.42, *t*_(48)_ = −0.465, *P* = 0.644, *b* = 0.14]. We observed a main effect of time on the accuracy [*B* = −0.13, SE = 0.03, *t*_(48)_ = −3.809, *P* < 0.001, *d* = 0.88], but not on the reaction time [*B* = 31.32, SE = 40.29, *t*_(48)_ = 0.777, *P* = 0.439, *d* = 0.16]. Both interactions between time and group were not observed [ACC: *B* = −0.03, SE = 0.47, *t*_(48)_ = −0.595, *P* = 0.554, *d* = 0.14; and RT: *B* = −55.34, SE = 56.98, *t*_(48)_ = −0.971, *P* = 0.334, *d* = 0.20] (see Figure 5).

#### Raven's Advanced Progressive Matrices (Far Transfer)

**Figure 6:**
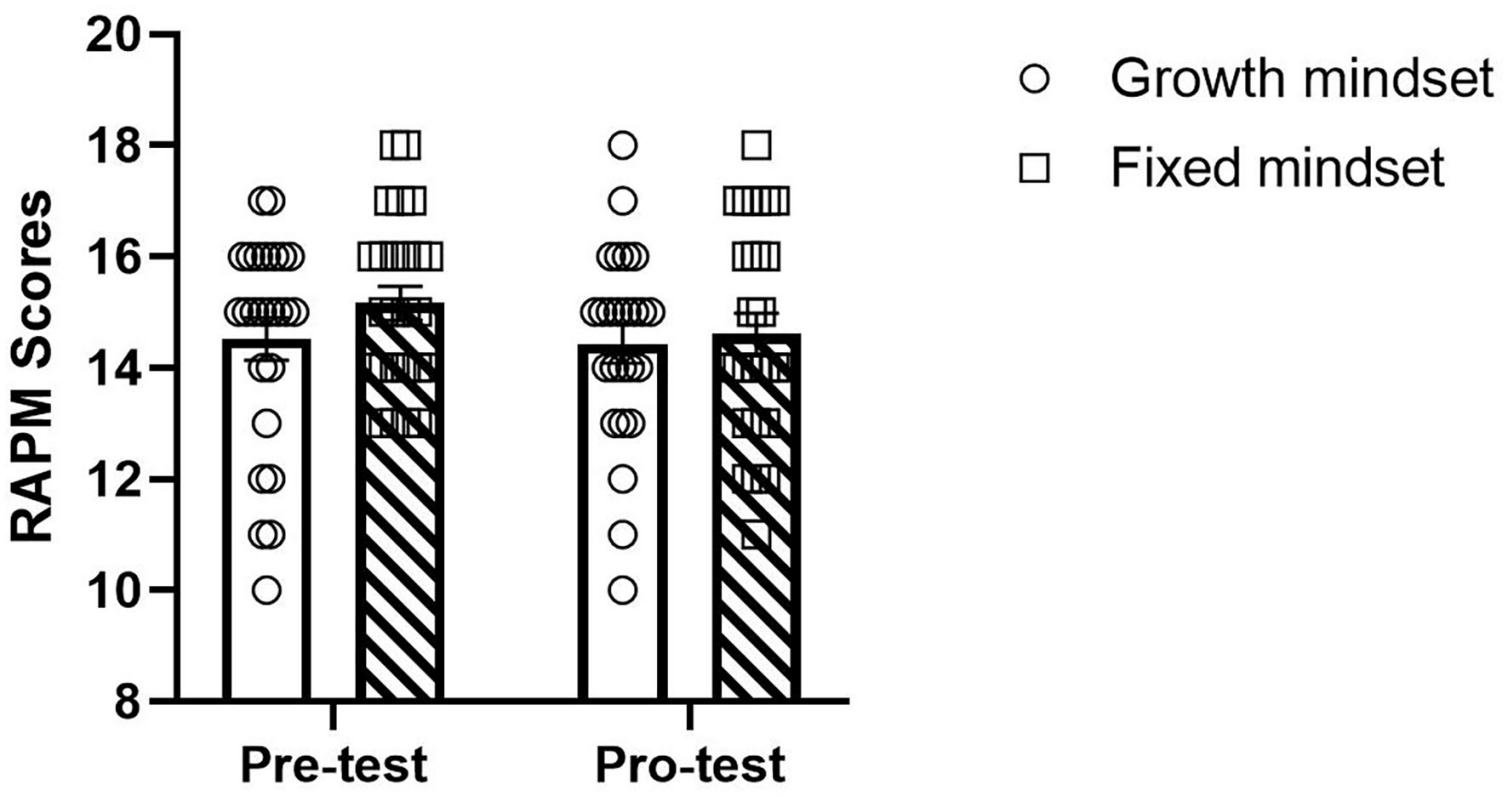
The difference between the fixed group and the growth group in Raven's Advanced Progressive Matrices (RAPM).

We analyzed the performance of RAPM between the two groups. The two groups did not differ in pretest [B = −64, SE = 0.50, *t*_(48)_ = −1.290, *P* = 0.203, *b* = 0.38) or post-test (*B* = −0.16, SE = 0.53, *t*_(48)_ = −0.304, *P* = 0.762, *b* = 0.09). We did not observe a main effect of time on test performance [*B* = 0.56, SE = 0.51, *t*_(48)_ = 1.095, *P* = 0.276, *d* = 0.22]. And there was not an interaction between time and group [*B* = −0.48, SE = 0.72, *t*_(48)_ = −0.664, *P* = 0.508, *d* = 0.14] (refer to Figure 6).

Overall, our data showed that the growth mindset group and the fixed group did not differ in training effects, near-transfer effect, and far-transfer effect.

## Discussion

Although evidence suggests that placebo effects exist in working memory training, no research has directly examined whether the mindset of intelligence is a critical variable. In this study, we replicated the protocol of the Foroughi et al.'s (2016) study that involved overt recruitment and a single, 1 h session of training. Importantly, we assigned participants to a growth mindset group and a fixed mindset group based on their TIS scores to avoid any confound. We tested for near-transfer effects using the 2-back task and far-transfer effects (fluid intelligence) using RAPM. However, the results showed that compared with the fixed mindset group, the performance of the growth mindset group was not significantly different from the pretest to the post-test in all tasks. It seems to suggest that mindset of intelligence does not contribute to the placebo effect in the 1 h working memory training.

Foroughi et al. (2016) recently attributed different TIS scores between two groups to their own selection of participants for the overt/covert recruitment, subjects who chose the overt flyer had higher expectancies and higher TIS scores. They ignored that the TIS score itself represents the mindset of intelligence that could cause the placebo effect, which means they confused the role of the mindset of intelligence and recruitment methods. Another possible explanation of Foroughi et al.'s (2016) study is that the results may be due to differences in the mindset of intelligence between the placebo group and the control group. Therefore, to our knowledge, this study is the first to directly test whether the mindset of intelligence is a contributor to placebo effects. However, the answer is no. It also triggers our deeper thinking about the mindset of intelligence. Compared with western students and ethnic Chinese students growing up in western countries, Chinese students have a more fixed mindset, which may be due to differences in reasoning about intelligence (Kim et al., 2017; Sun et al., 2021). This puts the relevant research into a broader cultural context and also suggests that we should not ignore the potential influence of social context, parenting variables, and educational style. Future studies should examine what variables influence the shaping of the mindset of intelligence of children and how cultural differences in the mindset of intelligence may lead to different outcomes. However, cultural differences are not directly responsible for the discrepancy between our results and those suggested by Foroughi et al. (2016). On the one hand, there was no difference between the TIS scores of both our two groups and their two groups. On the other hand, our finding is consistent with Thompson et al.'s (2013) study (a western study), which found that relevant cognitive factors such as mindset of intelligence have a negligible effect on training results and transfer effects.

We should note that we used a different training paradigm from Foroughi et al.'s (2016). However, this does not affect the results of the experiment. According to the response expectancy view (Kirsch, 1999), when studying placebo effects, setting a training task is only a cue to trigger the expectancy or motivation of participants. The improvement in performance due to the placebo effects should not depend on the training gains; that is why these studies chose an hour as training duration (no actual training gains). Besides, the three working memory training tasks in this study are also commonly used in cognitive training research (Zhao et al., 2011; Wang et al., 2014; Chen et al., 2018). Given this evidence, we believe that the difference shown in our research is not due to the different training paradigms. Another point worth noting is that we observed the main effect of time on the accuracy of the 2-back task. In view of the improvements in both groups, this might be a reflection of the practice effect, since the task was relatively easy for college students, and the interval was only an hour.

Overall, we found no evidence that there are placebo effects caused by the mindset of intelligence during working memory training. This study and Zhang et al.'s (2019) study reveal that neither the overt recruitment nor the mindset of intelligence contributes to placebo effects in 1 h working memory training. Two sets of explanations may account for these results.

First, do participants truly believe that 1 h of training can change their fluid intelligence? Just like researchers believe that rigorous and persistent cognitive training rather than 1 h cognitive training is practical, even the high TIS participants may think intelligence is malleable only with massive and long-duration practice; such a short training time is unrealistic. After all, the notion, fluid intelligence is hard to change, is well-known in researchers and laypeople. However, previous studies and this study ignored to examine actual expectancy of intelligence enhancement of participants in an hour, which should be addressed in future research. The goal of measuring the expectancy and motivation of participants in different intervention research stages is to infer the degree of engagement of participants in the training process (Tsai et al., 2018). The subjective report is usually used to evaluate expectancies and motivations. In the research of placebo effects, it is essential to evaluate the expectancy and motivation of participants before, during, and after the intervention. Measuring expectancy is the premise of examining its effect on positive training outcomes. However, it is unclear whether repeat measurement will expose the purpose of the experiment and weaken the expectancy or motivation of participants. Therefore, the appropriate approach to assess the expectancies and motivations needs to be further explored.

Second, can subjective expectancy improve the objective measurement of fluid intelligence? Pratkanis et al. (1994) found the illusory placebo effect: perceptions of personal improvement of participants were consistent with their expectations but inconsistent with objective measures. The illusory placebo effect also existed in working memory training, in which subjects believed that their cognitive abilities, such as intelligence, had been affected by the experiment in the absence of objective evidence (Redick et al., 2013). It challenges the placebo effect in intelligence research, which means IQ as a highly heritable ability (Plomin, 2004; Plomin et al., 2008; Sternberg, 2008) is hardly changed by subjective expectancies. To test this question persuasively, we suggest future cognitive training research to examine the relationship among expectancy, perceptions of change, and objective measurements of participants.

As far as we know, there are few studies on placebo effects in working memory training (Foroughi et al., 2016; Tsai et al., 2018; Zhang et al., 2019). Although these studies have some limitations, such as poor sample size, simple experimental design, and inconsistent results, we aimed to draw academic attention to placebo effects in cognitive training. The factors influencing the placebo effect are complex, and more empirical evidence is needed to promote the progress. Besides, the theoretical framework of placebo effects should be introduced. Previous studies lack theoretical depth in explaining the placebo effect phenomenon in working memory training and do not connect the findings with the broader field of placebo effect research. The main theoretical methods of studying placebo effects can be roughly divided into three views: classical conditioning, expectancy, and motivation (Geers et al., 2005). Interestingly, these three views are currently considered to be conflicting. Among them, the *response expectancy view*, which is widely mentioned in the perspective of expectancy, is a suitable model suggested by Foroughi et al. (2016). According to this theory, response expectancy is an automatic pre-reaction to situational and behavioral cues, a direct self-confirmation of individuals. The placebo effect is the direct and non-intermediary result of expectancy. In the study of Foroughi et al. (2016), the overt recruitment method was used to make the placebo group have stronger expectancy for the training results (intelligence can be improved); the working memory training task is only a cue to induce the placebo effect. If this theory holds, then when we use the same experimental design, no matter what type of working memory training task is used, it should always induce placebo effects. At present, the research on placebo effects in working memory training is not systematic and in-depth. We sincerely suggest that all researchers interested in this field should focus on absorbing beneficial inspiration from the classical theoretical model of placebo effects, which will promote our understanding of this field and further promote the progress of working memory training.

Although we failed to replicate the findings of Foroughi et al. (2016), we share their concern: researchers should pay more attention to the design of cognitive training experiments until substantial studies reveal the role of placebo effects. We suggest that participants should be assigned to one of three groups: training group, active control group, and no-contact control group. It is also necessary to measure the expectancies, subjective perceptions, and objective tasks of subjects. Cognitive abilities, especially fluid intelligence, can be improved is a promising finding for humans. On the one hand, we cannot exaggerate the training efficacy with placebo affecting actual training outcomes; on the other hand, we should not despise the training benefits just for concerns about placebo effects.

## Data Availability Statement

The raw data supporting the conclusions of this article will be made available by the authors, without undue reservation.

## Ethics Statement

The studies involving human participants were reviewed and approved by Ethical Evaluation of Research projects at the Department of Psychology—part of the School for Social and Behavioral Sciences at Nanjing University, China. The patients/participants provided their written informed consent to participate in this study.

## Author Contributions

XZ collected the data. PL and XZ analyzed the data and wrote the current version of the manuscript. RZ administrated the whole project. All authors contributed to the article and approved the submitted version.

## Funding

This study was financially supported by the National Defense Science and Technology Innovation Special Zone project of the Logistics Support Department of the Central Military Commission (Grant number 19-163-12-ZT-002-003-02) and the Fundamental Research Funds for the Central Universities (Grant number 2020300048).

## Conflict of Interest

XZ was employed by the company TCL Industries Holdings Co., Ltd. The remaining authors declare that the research was conducted in the absence of any commercial or financial relationships that could be construed as a potential conflict of interest.

## Publisher's Note

All claims expressed in this article are solely those of the authors and do not necessarily represent those of their affiliated organizations, or those of the publisher, the editors and the reviewers. Any product that may be evaluated in this article, or claim that may be made by its manufacturer, is not guaranteed or endorsed by the publisher.
